# Public spending for illegal drug and alcohol treatment in hospitals: an EU cross-country comparison

**DOI:** 10.1186/1747-597X-9-26

**Published:** 2014-06-30

**Authors:** Delfine Lievens, Freya Vander Laenen, Johan Christiaens

**Affiliations:** 1 Department of Accountancy and Corporate Finance, Ghent University, Sint-Pietersplein 7 9000, Ghent, Belgium; 2 Department of Penal Law and Criminology, Ghent University, Universiteitsstraat 4, 9000 Ghent, Belgium

**Keywords:** Drugs, Alcohol, Substance abuse, Public health, Hospital-based treatment, Europe, Public expenditure

## Abstract

**Background:**

In view of the current economic crisis and the resulting austerity measures being implemented by governments across Europe, public expenditure for substance abuse treatment has increasingly become a subject of discussion. An EU cross-country comparison would allow an estimation of the total amount of public resources spent on substance abuse treatment, compare various substance abuse treatment funding options, and evaluate the division of expenditures between alcohol and illegal drugs. The purpose of this study is to estimate the public spending of EU countries for alcohol and illegal drug abuse treatment in hospitals.

**Methods:**

Our study uses a uniform methodology in order to enable valid cross-national comparisons. Our data are drawn from the Eurostat database, which provides anno 2010 data on government spending for the treatment of illegal drug and alcohol abuse in 21 EU member states. The cross-country comparison is restricted to hospitals, since data were unavailable for other types of treatment providers. The systematic registration of in- and outpatient data is essential to monitoring public expenditures on substance abuse treatment using international databases.

**Results:**

Total public spending for hospital-based treatment of illegal drug and alcohol abuse in the 21 EU member states studied is estimated to be 7.6 billion euros. Per capita expenditures for treatment of illegal drug abuse vary, ranging from 0.1 euros in Romania to 13 euros in Sweden. For alcohol abuse, that figure varied from 0.9 euros in Bulgaria to 24 euros in Austria. These results confirm other studies indicating that public expenditures for alcohol treatment exceed that for illegal drug treatment.

**Conclusions:**

Multiple factors may influence the number of hospital days for alcohol or illegal substance abuse treatment, and expenditures fluctuate accordingly. In this respect, we found a strong correlation between gross domestic product (GDP) per capita and public expenditures per hospital day. The prevalence of problematic (illegal or legal) drug use in a country did not correlate significantly with the number of hospital days. Other factors must be included in the analysis of public expenditures for the treatment of substance abuse, such as the drug policy in a given country and the social norms regarding alcohol consumption.

## Introduction

Illegal drugs and especially alcohol have a significant health impact on human life in Europe. The burden of diseases resulting from alcohol and illegal drugs is enormous; together they account for 11% of disability adjusted life years (DALY’s^a^) lost in Europe [1]. The European Monitoring Centre for Drugs and Drug Addiction (EMCDDA) indicated that at least 1.2 million individuals received some kind of treatment for illegal drug use in the EU and its candidate countries [2]. In addition, Rehm, Shield, Rehm, Gmel & Frick [3] estimated that approximately 1.1 million people with an alcohol use disorder in the EU are in treatment^b^. A considerable share of substance abuse treatment is provided in hospitals. EU countries reported more than 161,000 hospital discharges for mental and behavioral disorders due to illegal drug use, and another 707,000 due to alcohol use in 2010 [4]. From the sheer number of people in treatment, it is clear that substance abuse treatment has an economic impact.

Rising health care costs have increased pressure on providers, insurers, and policymakers to monitor the costs of all health care services [5]. Moreover, public expenditures for substance abuse treatment are increasingly a subject of discussion in view of the economic crisis and of austerity. The cuts in government spending across Europe may affect substance abuse treatment; therefore, it is crucial that policymakers understand the economic value of substance abuse treatment services [6,7]. This economic evaluation of substance abuse treatment is gaining momentum [5], and the EU drugs strategy (2013–2020) supports this evolution by stating that actions must be evidence-based and cost-effective [8]. This puts a new premium on measuring and valuing the ‘return on investment’ of government expenditures for drug and alcohol abuse interventions [9]. This type of evaluation method is clearly linked to public expenditure studies, because it cannot be completed without the estimation of public spending on substance abuse treatment. Some aspects of drug and alcohol policy can be studied within a single country; for example, secondary school classrooms can be randomly assigned to receive one prevention curriculum or another, with effects on self-reported substance use assessed at follow-up. However, many important dimensions of policy operate at the national level, making cross-national comparisons of both policies and problem severity important.

Unfortunately, making valid cross-national comparisons can be surprisingly difficult because of differences in definitions, data, and organizational structures across countries. Creating a foundation for cross-national comparisons has been a multi-decade endeavor undertaken by many researchers, notably those at the European Monitoring Centre for Drugs and Drug Addiction (EMCDDA) [10]. This study contributes to that effort by estimating public expenditures on hospital-based treatment in a consistent manner for 21 EU member states.

The current study is unique in that few cross-national comparisons of substance abuse treatment costs have been conducted. A previous attempt to calculate the total European cost of illegal drug treatment services (published in a EMCDDA selected issue in 2011), suffered from limited data [6]. Rehm, Shield, Rehm, Gmel & Frick [3] estimated the social cost to the EU countries for the treatment and prevention of harmful alcohol use and alcohol dependence to be 6.3 billion euros (2010). However, they did not report on the cost for specific types of services such as outpatient treatment and inpatient treatment. As a result, the cost for hospital treatment is unknown. Neither is it possible to prorate the estimate of Rehm et al. [3] since the cost per episode of treatment tends to be higher in inpatient settings [6]. Our study aims to remedy this by estimating hospital-based treatment expenditures.

The study is conducted within the framework of policy evaluation and therefore focuses on public spending. Public expenditures are the direct instrument of public policy and they dominate in the financing of substance abuse treatment. Within the EU, health care is mainly financed by governmental funding because the public resources in the health care system are supported through general taxation and/or insurance-based systems [11].

Total public expenditures related to illegal drugs and alcohol (including but not limited to substance abuse treatment) have been estimated in social cost studies and public expenditure studies. Such studies have been conducted in Australia, the United States, Canada and some EU countries such as Belgium, Luxembourg, the Netherlands and Sweden [12-27]. While many of these studies follow a common set of general principles, they differ in particulars and so cannot support cross-national comparisons.

Some cross-country studies of costs or public expenditures in the field of alcohol and/or illegal drugs have been conducted in Europe, including for treatment spending [6,28-33]. However, while each of these studies are valuable, they suffer from one or more methodological problems. Kopp & Fenoglio [31] and the EMCDDA [6] were confronted with incomplete or imprecise data provided by the EU member states. Other studies merely compiled data from different national studies and so were confronted with data of varying quality [28-30]. Lievens et al. [32] even concluded that a truly valid cross-country comparison may be infeasible because of the conceptual and methodological differences in the national public expenditure studies.

Nevertheless, having an EU cross-country comparison of the public expenditures for substance abuse treatment would be valuable for several reasons [10,32,34]. It would allow one to estimate the total amount of public resources spent on substance use treatment. Moreover, it would allow comparison of substance abuse treatment funding in different countries. Country profiles providing information on treatment organization and its budgetary impact could be compiled and used as a first step in a full economic evaluation to find the most cost-effective way of organizing substance abuse treatment [9]. Finally, an EU cross-country comparison would enable examination of the division of expenditures between alcohol and illegal drugs, allowing for recommendations on resource allocations [35].

The remainder of this paper is organized as follows: first, public expenditures (including social security funds) for illegal drug and alcohol treatment in hospitals are presented and compared across EU countries. This will provide insight into the dynamics of substance abuse treatment organization across countries. Second, an estimate of the total EU spending on hospital substance abuse treatment is given. The public expenditures for illegal drug treatment are compared to expenditures for alcohol treatment. Finally, we discuss the factors that may influence the number of hospital days and the expenditures that come with it.

## Methods

Particular care was taken to ensure a uniform methodology across the EU member states studied in order to allow a valid cross-national comparison. Databases of international organizations were analyzed to identify health care expenditures for alcohol and illegal drug treatment^c^. The online databases of the following organizations were consulted: Organization for Economic Co-operation and Development (OECD); the European Commission; the World Health Organization (WHO); the United Nations (UN); European Monitoring Centre for Drugs and Drug Addiction (EMCDDA); European Medicines Agency (EMA); and European Centre for Disease Prevention and Control (ECDC). One would expect that these databases provide data on expenditures for various types of substance abuse treatment services. In the United States, both inpatient and outpatient cost of service groups include costs associated with mental health diagnosis, labs, and surgery services covered by Medicaid [7]. However, an analysis of the EU databases makes clear that Eurostat is the only database that provides consistent and comparable data for treatment provided in hospitals. Information for other types of treatment providers, such as nursing and specialized residential care facilities and providers of ambulatory health care are not consistently available. In view of this limitation, the current study focuses on hospital treatment.

The Eurostat database is used to measure public spending on illegal drug and alcohol treatment in hospitals. This database provides financial data (public health budgets for each type of treatment provider) with the System of Health Accounts^d^ and data on hospital activities (hospital days by diagnosis).

The financial data are collected by the System of Health Accounts (published by Eurostat, OECD and WHO), which systematically describes the financial flows related to health care [36]. For most EU countries, the public health budgets for each type of treatment provider are published on the Eurostat website [37], although there were no data available for six EU member states (Greece, Estonia, Ireland, Italy, Malta and the United Kingdom). In the System of Health Accounts, public health expenditures are identified as those labeled ‘the general government’, which includes the central, state and local government and social insurance funds. Hospital expenditures include the expenditures for general hospitals, for mental health and substance abuse hospitals, and for other specialty hospitals^e^ (e.g. hospitals for infectious diseases, rehabilitative and preventive services).

Eurostat also publishes hospital activities by diagnosis [38] for each country; aggregated data are provided for total hospital discharges and total hospital days. In theory, these hospital statistics cover the activities for general, mental health and specialty hospitals [E. Cayotte, personal communication, August 19, 2013], although six countries are not able to report the hospital days for all hospital types. Consultation of the Eurostat and WHO country metadata indicates that data are missing for Belgium, Denmark, France, Luxembourg, the Netherlands and Spain. Therefore, the results for the latter countries will be presented separately.

Based on the data in the Eurostat database, government spending on illegal drug and alcohol treatment in hospitals was identified using the following formula:

$$\begin{array}{ll} \text{average}\; & \text{cost}\;\text{per}\;\text{hospital}\;\text{day}\times \text{hospital}\;\text{days}\;\text{for}\;\\ & \text{treating}\;\text{illegal}\;\text{drug}\;\text{or}\;\text{alcohol}\;\text{disorders} \end{array}$$

This method has some limitations. The first limitation is that the average cost per hospital day is calculated by dividing the public health expenditure of hospitals by the total hospital days for treating all causes of diseases. This methodology assumes that all diagnoses have the same unit cost of treatment, despite the common-sense notion that the cost per hospital day varies across diagnosis. Furthermore, the hospital expenditures figure used to calculate the average cost per hospital day includes inpatient, emergency and outpatient services. The Eurostat database makes no distinction between types of treatment service. Consequently, the expenditures for outpatient and emergency services are attributed to inpatient activities and this leads to an overestimation of the average cost per hospital day. The second limitation is that the formula is based on the number of hospital days, but ‘hospital nights’ might be a more suitable term since hospital days are delineated as days in which a person admitted as an inpatient stays overnight in a hospital [39]. Thus, the measure excludes outpatient treatment and treatment of patients who were not admitted (e.g. those treated in the emergency room without admission). Nevertheless, economic cost studies frequently use hospital days to estimate the hospitals costs for treating substance abuse [15,16,21,27,40-43]. Hospital days are used as a measure because it is assumed to capture the prevalence of recorded substance abuse and they take into account the time spent for treatment. In the Eurostat database, hospital days with the primary diagnosis of mental and behavioral disorders due to psychoactive substance use or alcohol use (ICD10 codes^f^ F10-F19) are included. In the case of multiple diagnoses, the most severe and resource-intensive of these diagnoses is recorded as the primary diagnosis. Consequently, the public spending for substance abuse is underestimated because the patients with a non-substance-abuse-related primary diagnosis and a substance abuse disorder as secondary diagnosis are not taken into account. An overestimation is also possible for patients with a primary diagnosis of substance abuse and a secondary diagnosis (e.g. liver disease) that caused an extended stay in the hospital.

This cross-country comparison is conducted for 21 of the 27 EU member states^g^ with data anno 2010. The public expenditures for illegal drug and alcohol treatment in hospitals are reported per capita, as a share of gross domestic product (GDP) and total. In order to explain these results, the individual components of the formula (public expenditure per hospital day, hospital days for illegal drug or alcohol treatment, and the proportion of hospital days attributable to drug treatment) and prevalence rates are presented. Additionally, the total amount of public spending for the EU for illegal drug and alcohol treatment in hospitals is estimated. This estimation is restricted to 21 EU member states. An extrapolation to the EU-27^h^ is not possible given the lack of data on health expenditures^i^ for six EU member states (Greece, Estonia, Ireland, Italy, Malta and the United Kingdom).

## Results

### Public spending for illegal drug treatment in hospitals

#### ***Numeric results***

Public expenditures for hospital treatment are presented in two tables. Table 1 presents the countries that register the illegal drug treatment hospitals days and expenditures for all types of hospitals (general, mental health and specialty hospitals). The countries in Table 2 only provide data for general (2 countries) and specialty (4 countries) hospitals.

**Table 1:** Hospital days and expenditures for illegal drug treatment (general, mental health and specialty hospitals), for 15 EU countries, 2010

Country^*^	**Public expenditure per hospital day (euros)**	**Hospital days for illegal drug treatment per 1,000 capita**	**Proportion of hospital days attributable to illegal drug treatment (%)**	**Illegal drug treatment expenditure by hospitals (million euros)**	**Illegal drug treatment expenditure by hospitals, per capita (euros)**	**Illegal drug treatment expenditure by hospitals, as percentage of GDP**
Sweden	1532	9	0.88%	123	13.2	0.035%
Austria^†^	507	15	0.62%	65	7.8	0.023%
Germany	391	16	0.72%	523	6.4	0.021%
Slovenia	432	7	0.59%	6	3.2	0.018%
Finland^†^	428	6	0.28%	14	2.5	0.008%
Slovakia	165	11	0.75%	9	1.7	0.014%
Poland	167	9	0.70%	55	1.4	0.015%
Czech Republic	211	17	0.79%	37	3.5	0.025%
Portugal	1045	0.6^‡^	0.11%	6	0.6	0.004%
Hungary	121	5	0.28%	6	0.6	0.006%
Latvia^†^	140	3	0.24%	0.8	0.4	0.005%
Bulgaria	69	3	0.19%	2	0.2	0.004%
Lithuania	113	1	0.06%	0.4	0.1	0.001%
Romania	81	1	0.07%	2	0.1	0.002%
Cyprus^†^	936	0.01^‡^	0.002%	0.006	0.01	0.00003%
**Mean**^k^**(SD)**	**423 (429)**	**7 (6)**	**0.42% (0.31%)**	**57 (133)**	**2.8 (3.7)**	**0.012% (0.01%)**

^*^The European countries are not classified in regions, because no global classification system is available for illegal drugs (contrary to studies on alcohol, which distinguish geographical areas by drinking traditions and patterns [44]). Drug-related research e.g., ([45,46]) uses different types of classification according to the investigated type of drugs. Nevertheless, the conclusions for multiple countries are described by the UN geographical regions: Eastern Europe, Northern Europe, Southern Europe and Western Europe.

^†^Contrary to the other countries, the live-born infants (Z38) of Austria, Cyprus, Latvia and Finland are not included in the total hospital days, and this could lead to an overestimation of the hospital expenditures.

^‡^The data for Cyprus cover only public sector hospitals. Portugal covers all public inpatient institutions and only two private hospitals. Consequently, the hospital days for illegal drug treatment are underestimated, and this may affect the proportion of hospital days attributable to illegal drug treatment. Therefore, the hospital days and public expenditures for illegal drug treatment in Cyprus and Portugal will not be further analyzed.

**Table 2:** Hospital days and expenditures for illegal drug treatment (general and specialty hospitals), for 6 EU countries, 2010

**Country**	**Public expenditure per hospital day (euros)**	**Hospital days for illegal drugs per 1,000 capita**	**Proportion of hospital days attributable to illegal drug treatment (%)**	**Illegal drug treatment expenditure by hospitals (million euros)**	**Illegal drug treatment expenditure by hospitals, per capita (euros)**	**Illegal drug treatment expenditure by hospitals, as percentage of GDP**
	**General hospitals**					
France	1036	0.4	0.04%	29	0.4	0.001%
Denmark^*^	2125	0.2	0.02%	2	0.4	0.001%
**Mean (SD)**	**1580 (770)**	**0.3 (0.2)**	**0.03% (0.02%)**	**15 (19)**	**0.4 (0.06)**	**0.001% (0.0004%)**
	**General and Specialty hospitals (no mental health or substance abuse hospitals)**					
Luxembourg^†^	1079	32	2.50%	17	34	0.044%
Spain^†^	1131	2	0.27%	97	2.1	0.009%
Belgium^*^	579	2	0.16%	12	1.1	0.003%
Netherlands	1620	0.5^d^	0.07%	12	0.7	0.002%
**Mean (SD)**	**1102 (426)**	**9 (15)**	**0.75% (1.17%)**	**35 (42)**	**9.6 (16.6)**	**0.015% (0.02%)**

^*^Denmark and Belgium have only data with hospitals days available for 2009.

^†^Contrary to the other countries, the live-born infants (Z38) of Luxembourg and Spain are not included in the total hospital days, and this could lead to an overestimation of the hospital expenditures.

Table 1 shows that on average the hospital expenditure for illegal drug treatment in the EU-15 is 2.8 euros per capita and 0.01% of GDP. Table 1 also shows important differences between EU countries. Sweden invests the most in hospital-based illegal drug treatment (per capita 13 euros and 0.035% of GDP), primarily because its costs per hospital day appear to be extremely high. Austria (per capita 8 euros and 0.023% of GDP) and Germany (per capita 6 euros and 0.021% of GDP) complete the top three, combining fairly high hospital expenditures (per capita)^j^ with high rates of hospital-based treatment for illegal drugs. A number of Northern and Eastern European countries (Bulgaria, Hungary, Romania, Lithuania and Latvia) reported hospital expenditures lower than 1 euro per capita and 0.01% of GDP. The cost per hospital day of these countries is less than one-third of the average in Sweden, Austria, and Germany, and this is combined with rates of hospital-based treatment per capita that are less than one-sixth in size.

As discussed above, France and Denmark only provide data for general hospitals. Belgium, Luxembourg, the Netherlands and Spain have information for specialty hospitals and general hospitals, but not for mental health hospitals. Therefore, the expenditure estimates for the countries in Table 2 are obviously underestimated. Nonetheless, Table 2 shows that on average the hospital expenditure for illegal drug treatment in the EU-4 is 9.6 euros per capita and 0.015% of GDP. Luxembourg has the highest share of hospital days (2.50%) and of expenditures (34 euros per capita) attributable to illegal drugs in the EU. This could be explained by its relatively high public expenditures for hospital care and prevalence of problem drug use (6.2 per 1.000 capita) [47].

#### ***Interpretation***

The hospital expenditures for illegal drug treatment are calculated on the basis of public health expenditures and hospital days.

First, the average public expenditure per hospital day across the 15 EU countries is 423 euros per day. Countries in Western and Southern Europe (except Germany) spend more than this average, with Sweden reporting the highest expenditure (1532 euros). Countries in Eastern Europe reported much less public funding in hospitals. The lower expenditures of Eastern European countries are mainly due to the lower economic power in terms of GDP [48] and the lower proportion of public financing of health expenditures [49].

Second, the number of hospital days for illegal drug treatment per 1,000 capita range from 1 to 17 days. Austria (15), Germany (16) and the Czech Republic (17) registered the highest number of hospital days, whereas Lithuania (1) and Romania (1) registered the least number of hospital days. When comparing hospitals days for illegal drug treatment to the total number of hospital days, Sweden (0.88%), Slovakia (0.75%), Germany (0.72%) and the Czech Republic (0.79%) have the largest proportion of hospital days for illegal drug treatment. This may indicate that the latter countries organize drug treatment mainly inside hospitals and/or that they are confronted with a high number of problem drug users.

**Figure 1:**
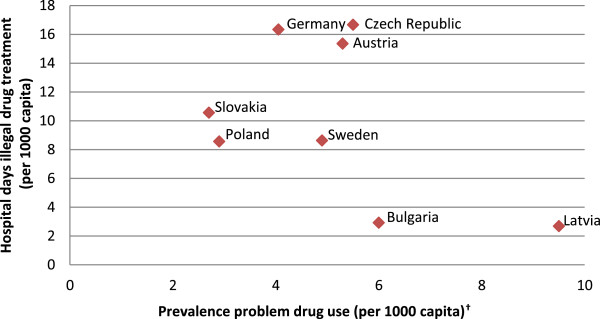
Prevalence of problem drug use (2007-2011^*^) versus hospital days for 8 EU countries. ^*^The prevalence for problem drug users aged 15–64. Depending on the availability of data, prevalence estimates are presented for the years 2007, 08, 09, 10 or 11. ^†^The EMCDDA did not provide prevalence rates for Slovenia, Finland, Portugal, Hungary, Lithuania and Romania.

To discover whether differences in the prevalence of problem drug users can explain the observed (differences in) hospital days, Figure 1^l^ plots the number of hospital days used for drug treatment versus the number of problem illegal drug users (12 months prevalence), both expressed in per capita terms using data from the statistical bulletin of the EMCDDA [47].Pearson’s correlation coefficient was used to analyze the linear association between hospital days and the prevalence of problem drug use, however no positive correlation is observed (r = -0.468, p = 0.243). This implies that the high number of hospital days in Sweden, Slovakia, Austria and Germany cannot be explained by these country’s prevalence rates of problem drug use. However, Figure 1 gives an indication of how drug treatment is organized in the 8 EU member states. On the one hand, Germany, Austria, Slovakia, Poland and the Czech Republic have a high number of hospital days in comparison with the prevalence of problem drug use. In these countries, problem drug users treated in a hospital stayed three-to-four days on average. On the other hand, Latvia and Bulgaria report less than one hospital day per problem drug user despite their having a high prevalence of problem drug use. It seems that most problem drug use treatment in these countries is organized outside hospitals. An alternative explanation could be that these countries provide less treatment altogether.

### Public spending for alcohol treatment in hospitals

#### ***Numeric results***

Results for public expenditures for alcohol treatment in hospitals are also presented in two separate tables (Table 3 and Table 4) depending on whether data were available for the different types of hospitals. The countries are divided into four geographical areas based on the WHO classification concerning drinking traditions and patterns [44]. However, it is difficult to draw conclusions on a regional level due to missing data for 12 EU member states (Belgium, Denmark, Estonia, France, Greece, Ireland, Italy, Luxembourg, Malta, Netherlands, Spain and the United Kingdom).

**Table 3:** Hospital days and expenditures for alcohol treatment (general, mental health and specialty hospitals), for 15 EU countries, 2010

**Country**	**Public expenditure per hospital day (euros)**	**Hospital days for alcohol treatment per 1,000 capita**	**Proportion of hospital days attributable to alcohol treatment (%)**	**Alcohol treatment expenditure by hospitals (million euros)**	**Alcohol treatment expenditure by hospitals, per capita (euros)**	**Alcohol treatment expenditure by hospitals, as percentage of GDP**
**Central-western and western country group (Belgium, France, Ireland, Luxembourg, Netherlands and UK missing)**						
Austria^*^	507	47	1.88%	198	23.7	0.069%
Germany	391	49	2.16%	1578	19.3	0.063%
Mean (SD) Central-western and western country group	449 (82)	48 (2)	2.02% (0.2%)	888 (976)	21.5 (3.1)	0.066% (0.004%)
**Central-eastern and eastern country group (Estonia missing)**						
Slovenia	432	34	2.69%	30	14.5	0.084%
Slovakia	165	58	4.11%	51	9.5	0.078%
Poland	167	51	4.15%	325	8.5	0.092%
Hungary	121	31	1.76%	38	3.8	0.039%
Latvia^*^	140	19	1.68%	6	2.7	0.033%
Czech Republic	211	53	2.54%	118	11.3	0.079%
Lithuania	113	19	1.08%	7	2.2	0.026%
Romania	81	13	0.73%	22	1.0	0.018%
Bulgaria	69	13	0.87%	7	0.9	0.019%
Mean (SD) central-eastern and eastern country group	166 (109)	32 (18)	2.18% (1.30%)	67 (103)	6.0 (5.0)	0.052% (0.030%)
**Nordic countries (Denmark missing)**						
Sweden	1532	11	1.10%	155	16.6	0.044%
Finland^*^	428	25	1.19%	57	10.7	0.032%
Mean (SD) nordic countries	980 (780)	18 (10)	1.14% (0.06%)	106 (69)	13.6 (4.1)	0.038% (0.009%)
**Southern Europe (Greece, Italy, Malta and Spain missing)**						
Portugal	1045	3^†^	0.49%	29	2.7	0.017%
Cyprus^*^	936	0.03^†^	0.01%	0.1	0.1	0.001%
Mean (SD) southern Europe	990 (78)	1.3 (1.8)	0.25% (0.35%)	14 (20)	1.4 (1.8)	0.009% (0.011%)
**Mean (SD) EU-15**	**423 (429)**	**28 (19)**	**1.76% (1.21%)**	**175 (399)**	**8.5 (7.4)**	**0.046% (0.029%)**

^*^The live-born infants (Z38) of Austria, Cyprus, Latvia and Finland are not included in the total hospital days, and this could lead to an overestimation of the hospital expenditures.

^†^The data of Cyprus covers only public sector hospitals. Portugal covers all public inpatient institutions and only two private hospitals. Consequently, the hospital days for alcohol treatment are underestimated, and this may also affect the proportion of hospital days attributable to alcohol treatment. The results of Southern Europe will not be analyzed due to this missing data.

**Table 4:** Hospital days and expenditures for alcohol treatment (general and specialty hospitals), for 6 EU countries, 2010

**Country**	**Public expenditure per hospital day (euros)**	**Hospital days for alcohol treatment per 1,000 capita**	**Proportion of hospital days attributable to alcohol treatment (%)**	**Alcohol treatment expenditure by hospitals (million euros)**	**Alcohol treatment expenditure by hospitals, per capita (euros)**	**Alcohol treatment expenditure by hospitals, as percentage of GDP**
	**General hospitals**					
Denmark^*^	2125	3	0.40%	39	7.1	0.017%
France	1036	5	0.52%	331	5.1	0.017%
**Mean (SD)**	**1580 (770)**	**4 (1)**	**0.46% (0.08%)**	**185 (206)**	**6.1 (1.4)**	**0.017% (0.0003%)**
**General and Specialty hospitals (no mental health or substance abuse hospitals)**						
Luxembourg^†^	1079	76	5.94%	41	81.9	0.105%
Belgium^*^	579	9	0.72%	53	4.9	0.015%
Spain^†^	1131	3	0.38%	143	3.1	0.014%
Netherlands	1620	1	0.19%	34	2.1	0.006%
**Mean (SD)**	**1102 (426)**	**22 (36)**	**1.81% (2.76%)**	**68 (51)**	**23 (39.3)**	**0.035% (0.047%)**

^*^Denmark and Belgium have only data with hospitals days available for 2009.

^†^The live-born infants (Z38) of Luxembourg and Spain are not included in the total hospital days, and this could lead to an overestimation of the hospital expenditures.

Table 3 shows that on average the hospital expenditure for alcohol treatment in the EU-15 is 8.5 euros per capita and 0.046% of GDP. We see important differences between EU countries. The Central-Western and Western country group have the highest expenditures in terms of GDP (average of 0.066%) and per capita with 24 euros for Austria and 19 euros for Germany. This contrasts with many members of the Central-Eastern country group that reported an average hospital alcohol treatment spending of 6 euros per capita (0.052% of GDP). The Nordic country group reported spending of 14 euros per capita but the lowest expenditures in terms of GDP (0.038%).

Table 4 provides an overview of the six countries that provided data limited to general (and specialty) hospitals. As was the case for Table 2, the expenditures in hospitals in the countries in Table 4 are underestimated. Despite the lack of data from mental health hospitals, on average the hospital expenditure for alcohol treatment in the EU-4 is 23 euros per capita and 0.035% of GDP. Luxembourg has the highest hospital expenditure (82 euros per capita and 0.1% of GDP) for alcohol treatment in the EU, and the share of hospital days (5.94%) attributable to alcohol is high in comparison with the other EU countries (1.81%). At first sight, this could not be explained by the prevalence of people with alcohol dependence (which is relatively low; 3.4%) [3].

#### ***Interpretation***

In this section we explain hospital expenditures for alcohol treatment using the financial investment in public health and hospital use.

The first conclusion is that the public expenditures per hospital day vary extensively ranging from 69 euros in Bulgaria to 1532 euros in Sweden. The average in Eastern European countries of 166 euros is much lower than the average of the 15 countries studied (423 euros). Consequently, countries with a similar proportion of hospital days attributable to alcohol treatment (e.g. Sweden and Lithuania) could have a different outcome in terms of alcohol treatment expenditure per capita. As is the case for expenditures on illegal drug treatment, the lower expenditures of Eastern European countries could be explained by the mix of public and private funding of health care [49] and differences in terms of GDP [48].

**Figure 2:**
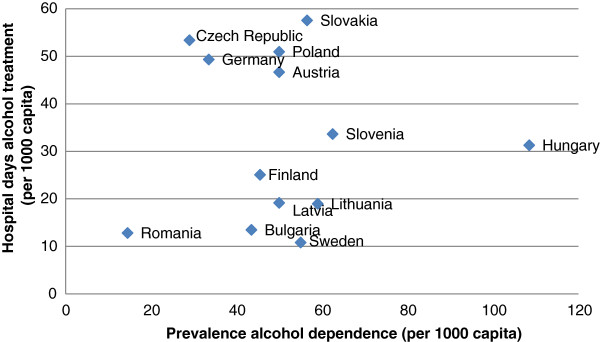
Prevalence of alcohol dependence (1999-2009^*^) versus hospital days for 13 EU countries. ^*^The prevalence of men and women aged 18–64. Depending on the availability of data, prevalence estimates are presented for varying years.

The second conclusion is that Slovakia, Poland and the Czech Republic reported the highest number of hospital days for alcohol treatment, with more than 50 hospital days per 1,000 capita, a rate that translates to alcohol treatment accounting for more than 4% of hospital days in Slovakia and Poland. The Central-Western and Western country group reported on average 48 hospital days per capita (2.02% of hospital days for alcohol treatment). In the Nordic countries, the number of hospital days for alcohol treatment is limited to 1.14%. As is the case for illicit drugs, the hospital days are investigated by looking at a country’s substance abuse treatment organization and the prevalence rates. Rehm et al. [3] provide an overview of 12-month prevalence rates for alcohol dependence per European country. In Figure 2, these prevalence rates are compared with the hospital days for alcohol.

As was the case for illegal drugs, no significant Pearson correlation (r = 0.003, p = 0.991) was found between hospital days and the prevalence of alcohol dependence. The majority of European countries (8) have a prevalence of alcohol dependence between 4% and 6%. An exceptional case is Hungary, which has the highest prevalence rate of alcohol dependence with 10.85%, although the Hungarian number of hospital days for alcohol treatment lies below the average of the 13 countries studied (32.5 days). It is likely that Hungary, and Sweden and Bulgaria as well, organize alcohol treatment mostly outside of hospitals because for an estimated average of three-to-five persons with alcohol dependence [3] only one hospital day is recorded in these countries. By contrast, Germany, Slovakia, Poland and the Czech Republic reported more than one hospital day of treatment per person with alcohol dependence.

#### ***Comparing public spending in the EU-21 for illegal drug and alcohol treatment in hospitals***

Public spending in the EU-21 for illegal drug and alcohol treatment in hospitals is presented in Table 5. The public spending of six EU member states (Belgium, Denmark, France, Luxembourg, the Netherlands and Spain) with data limited to general and/or specialized hospitals is extrapolated to all types of hospitals. To do this, we pro-rated total hospital expenditures of the six EU countries in proportion to the number of patient days associated with substance abuse treatment. Tables 1 and 3^m^ provide the proportion of hospital days attributable to illegal drug and alcohol treatment. The weighted average of the 15 EU countries, based on population, is 0.56% for illegal drugs and 2.18% for alcohol.

**Table 5:** Public expenditures for illegal drug and alcohol treatment in hospitals for 21 EU countries^*^, 2010

	**Public expenditures (million euros)**	**Public expenditures per capita (euros)**	**Public expenditures in % of GDP**
Illegal drug treatment	1,703	4.7	0.020%
Alcohol treatment	5,930	16.5	0.069%
**Total**	**7,633**	**21.2**	**0.089%**

^*^Source for population and GDP of EU countries: Eurostat [50,51].

Table 5 shows that the total EU-21 spending for illegal drug and alcohol treatment in hospitals is estimated to be 7,633 million euros or 21 euros per inhabitant; the share of GDP is 0.089%. The hospital expenditures for alcohol treatment are three times higher than the expenditures for illegal drug treatment, due to the higher number of hospitalization days for alcohol treatment (Tables 1 and 3 report 28 hospital days for alcohol treatment per 1,000 capita as compared to 7 days for illicit drug treatment).

## Discussion

This cross-country comparison provides insight into the public spending of governments in the EU on substance abuse treatment in hospitals. A uniform methodology based on international databases is used to provide consolidated data on the public expenditures for drug and alcohol treatment in hospitals. The total public spending on hospital-based substance abuse treatment is estimated to be 7.6 billion euros in the 21 EU countries. Three-quarters (77.7%) of these public expenditures are used for alcohol treatment, while the remaining quarter (22.3%) is used for illegal drug treatment. That public spending for alcohol treatment exceeds spending for illegal drug treatment is consistent with previous studies e.g. [17,26,27]. As expected, the estimate of 5.9 billion euros in public expenditures for alcohol treatment in hospitals is lower than the estimate of Rehm et al. [3]. They estimated a European cost, including private expenditures, of 6.3 billion euros for alcohol treatment and prevention. It is difficult to compare these studies and draw conclusions given the lack of data for all EU member states. This points to the importance of an international database with complete data for all EU countries. In the United States, data collection on the nominal costs of billed services attached to each individual client under, e.g., Medicaid is standardized across the fifty states. That is not the case in Europe [7].

This study also showed a large variation in public spending on substance abuse treatment in hospitals across the 15 EU member states that did provide comparable data. These results are discussed by looking at the explaining factors, the policy implications and limitations of the study.

### Explaining factors

The public spending on hospitalized substance abuse treatment can be explained by a variation in three factors: 1) the hospital cost per day, 2) the organization of substance abuse treatment and 3) the prevalence of problem illegal drug use and alcohol dependence. We elaborate on each in turn.

*The hospital cost per day* is influenced by the structure of health care expenditures. The health expenditures in Central‒East and Eastern Europe are much lower than in the other EU countries, because these countries have lower GDP per capita [48]. There is a strong (Pearson) correlation between GDP per capita and the public expenditures per hospital day (r = 0.638, p = 0.002). Next, the mix of public and private health financing may help explain differences in *public* spending on health care [see Additional file 1]. Most Eastern European countries are characterized by a limited share of public financing: 56% in Bulgaria, 64% in Hungary and 68% in Slovakia (one exception is 83% in the Czech Republic) [52]. The economic crisis affected the public-private financing mix for countries such as Bulgaria and Slovakia, since they reported a substantial increase of the private contribution and a corresponding decrease in public expenditure in 2010 [53]. It is very likely that Denmark, Sweden and the Netherlands reported the highest public expenditures per hospital day partly because their proportion of public financing is high: the Netherlands 86%, Denmark 85% and Sweden 82%. Moreover, in Eastern Europe it appears that informal patient payments continue to exist despite reforms within the health care sector [54,55]. Private health insurance and out-of-pocket expenditures have a negative impact on the accessibility to health care, and this is linked to the high share of private financing [56]. Limited accessibility may lead to an additional limitation of the number of hospital days.

Moreover, public hospital expenditures are influenced by the source of financing, i.e. general taxation or insurance-based systems. Countries with predominantly insurance-based systems (e.g. Belgium, France, the Netherlands, Germany and Luxembourg) have higher health care expenditures, because the insurance-based system is characterized by a lower degree of control over expenditures [11].

The number of hospital days directly influences public spending for alcohol and drug treatment. These hospital days are in turn influenced by the *organization of substance abuse treatment* in a country [57]. The Western European countries Austria and Germany reported a high number of hospital days per capita attributable to alcohol and illegal drug treatment. The Eastern European countries Poland and Slovakia also reported high hospital days, especially for alcohol treatment. This high number of hospital admissions/days suggests that these countries organize drug treatment mainly inside hospitals, while countries with a low number of hospital days may have more of a tradition of establishing specialized treatment outside hospitals. However, an alternative explanation is that the latter countries have shorter hospital stays. In fact, a couple of countries with a low number of alcohol hospital days reported a shorter average in-patient length of stay for alcohol treatment (e.g. Romania: 11.3 days, Lithuania: 8.3 days and Sweden: 4.7 days) than Austria (17.6 days) and Germany (13.3 days). The same conclusion can be drawn for illegal drug treatment, except for Bulgaria which reported longer stays for alcohol (27.6 days) and illegal drug treatment (18 days) [58].

Furthermore, the profile and the preferences of substance abusers may also influence the organization of drug treatment. Substance abusers have a personal preference for a specific type of treatment service that is based on indicators such as flexibility, accessibility, proximity of treatment service, etc. [59,60]. Moreover, clients may prefer outpatient treatment because it entails fewer out-of-pocket expenses. Outpatient treatment allows female clients to continue caring for their children [61].

In addition to the hospital cost per day and the organization of substance abuse treatment, we investigated whether *the prevalence of illegal drug and alcohol problems* in a country can explain the number of hospital days and the expenditures that come with it. One might expect that the more a country is confronted with substance abusers, the higher the hospital occupation for these problems will be. In fact, this is the presumption of economic-cost studies using hospital days to estimate the hospitals costs for treating substance abuse. However, we found no positive correlation between these two variables. For example, Latvia and Bulgaria reported a high prevalence of problem drug use in combination with a low number of hospital days. In this respect, the way a country’s drug treatment is organized influences the relation between prevalence of substance abuse and number of hospital days. Furthermore, the prevalence rates could be affected by cultural factors and social norms regarding substance use. Rehm et al. [62] argue that alcohol is highly culturally embedded in Southern-European countries, therefore people in the region are more likely to deny alcohol dependence and this may result in lower admissions to hospitals. This shows that monitoring (trends in) the prevalence of problem drug users will not suffice to monitor the (trends) in public expenditure on substance use treatment.

It should be noted that the expenditures on alcohol and illicit drug treatment cannot be explained solely by looking at the combination of the prevalence of problem illegal drug use and alcohol dependence, the hospital cost per day and the organization of substance abuse treatment in a country. Other factors such as a country’s cultural and social norms regarding substance use, its illicit drug or alcohol policy or the labor costs could play a role as well [6]. We identified an impact of these factors for the two outliers of this EU cross-country comparison. First, Sweden was the outlier in public spending for illegal drug treatment. Its public expenditure of 13 euros per capita can be explained by the high cost of hospitalization and the high proportion of hospital days attributable to illegal drug treatment (see Table 1). Sweden’s drug policy may be an additional explanatory factor since Sweden prioritizes a drug free society and abstinence-driven treatments [63]. This approach may also be more expensive than other drug treatment policies. Cost-benefit analyses need to be consulted to determine which drug treatment investments bring about (financial) gains. Second, Luxemburg spends the most in Europe per capita on hospital expenditure for both alcohol and illegal drug treatment. The prevalence of problem illegal drug use (6.2 per 1.000 capita) [47] and the high proportion of drug clients entering inpatient centers (79%) [64] influences the number of hospital days for illicit drugs. For alcohol, the share of hospital days could not be explained by the prevalence of people with alcohol dependence. We speculate that the high expenditures for Luxembourg could be ascribed to the smaller scale of drug treatment organization that imposes more costs on the health care budget. These examples demonstrate that multivariate research is necessary to determine which factors affect public spending on substance abuse treatment.

### Policy implications

This study measured how much European governments spend treating illicit drug and alcohol problems in hospitals. Governments play an important role in financing health care, since governments in the EU-21 finance on average 73% of the health expenditures (see Additional file 1). This differs from the United States where less than 50% of health spending is publicly financed. We would like to highlight the importance of measuring direct treatment costs in political and policy decision-making. The comparability of results across countries provides information for policymakers and public administration [10]. The impact of substance abuse treatment in hospitals on a country’s budget is presented, and these data can be used to illustrate the budgetary consequences of different drug policies. The cost information in this study also provides a valuable basis for assessing total public spending on substance abuse treatment, not just hospital-based spending. It can also contribute to the evaluation of substance abuse interventions [65,66], since the public expenditure studies provide an important component for economic evaluation studies: the public expenditures serve as the independent variable and outcomes (e.g., OD deaths) as the dependent variable. Moreover, these economic evaluation studies can be used to conduct more complete economic evaluations of substance abuse treatment in EU countries. For example, country profiles could be developed compiling information on treatment organization and budgetary impact. Ideally, these efforts lead to an evidence-based policy where financial resources are assigned to cost-effective substance abuse treatment [67]. However, it remains to be seen if governments will be willing and able to make these investments in exchange for benefits in the long-term (i.e. cost savings and reduced human suffering). For example, Rehm et al. [3] estimated that less than 10% of people with alcohol dependence in the EU receive treatment^n^. A 10% increase in health care coverage for hospital-based alcohol treatment in Europe would bring about an estimated 593-million-euro increase in hospitals’ public expenditure.

### Study limitations and recommendations

This study uses data from the Eurostat database to measure how much European governments spend on treating illegal drug and alcohol problems in hospitals. International databases facilitate cross-country comparisons that could highlight the impact of substance abuse on public health budgets [68]. Our cross-country comparison is restricted to hospitals since data were unavailable for other types of treatment providers. It is not clear which proportion of the drug and alcohol clients receive hospital treatment. The Treatment Demand Indicator (TDI^o^) used in the EU, cannot determine the proportion of substance use clients treated in hospitals since it only distinguishes between the proportion of illegal drug clients in inpatient^p^ and outpatient centers. The TDI shows that the proportion of reported clients entering inpatient centers for drug-related problems varies to a large extent by country (from 2% in France to 79% in Luxembourg) [64]^q^. Notwithstanding the limitations of the current analysis, the impact of hospital expenditures for drug and alcohol treatment on the public budget should not be underestimated. Multiple studies e.g. [6,69,70] show that the unit cost for hospital treatment is much higher than for outpatient treatment services. For example, inpatient detoxification in England is provided at a cost of 200 euros per patient per day and outpatient detoxification is provided at a cost of 8 euros per patient per day [71]. Moreover, Andlin-Sobocki, Jönsson, Wittchen and Olesen [72] indicate that the cost for hospital care due to brain disorders in Europe (including alcohol and illicit drug use disorders) dominates total treatment cost. Public expenditure studies indicate that a large share of the public expenditures for substance abuse treatment is attributable to care in hospitals. For example, in Belgium the share of hospital treatment for alcohol and illegal drug use amounts to as much as 90.66% of the total public spending for substance abuse treatment [21]. On the other hand, a Swedish study, with a research scope limited to illegal drugs, found a much lower share of hospital treatment. Ramstedt [14] reported that hospital expenditures made up only 16% of the total illegal drug abuse health spending. In other words, insight into the expenditures on substance abuse treatment via hospital expenditures is complex, since it varies with the investigated substance and with several other factors discussed above.

The analysis of international data for cross-country comparison purposes illustrated that, despite the great potential of these data (bases), much information is still lacking today. Ideally, these databases should provide hospital charges categorized according to diagnosis-related groups, as is the case with the Medicaid database in the United States [7]. However, the Eurostat database is limited to public health care expenditures by provider (e.g. ambulatory health care, nursing and residential care facilities). In order to estimate the drug- and alcohol-related percentages of these budgets, the health care activities by diagnosis are required for outpatient and inpatient treatment services (apart from hospital-based treatments). Further research is necessary to develop variables in international databases that provide data for outpatient treatment sessions for substance abuse, inpatient days for substance abuse, consultations for substitution treatment, drug treatment counseling in prisons, etc. This data would allow researchers to compare public expenditures for different types of treatment regimes. Additionally these data could be used for more in-depth economic evaluations, i.e. whether specific treatment modalities are more cost-effective than others [73,74].

In our study in particular, we were confronted with the significant limitations of the Eurostat database. In the Eurostat database, hospital days are limited to primary diagnosis and health expenditures are not subdivided by inpatient, emergency or outpatient service.

Next, the Eurostat data are sometimes incomplete because countries are not always able to provide data for all types of hospitals (general, mental health and specialty hospitals). A number of EU countries (Belgium, Denmark, France, Luxembourg, the Netherlands and Spain) could not report data for mental health hospitals. Furthermore, the external causes of morbidity (the ICD-10 codes V00-Y84), such as accidents, intentional self-harm and assault [75] are not included in the total hospital days, resulting in a higher proportion of hospital days attributable to drugs/alcohol. This in turn leads to an *overestimation* of hospital expenditures for alcohol and illicit drug treatment in hospitals.

Finally, Eurostat collects health care data via various public and private information sources in EU countries. These data reflect the country-specific way of organizing and reporting health care, and this may diminish comparability across countries [76]. In this respect, the health expenditures collected by the System of Health Accounts (SHA) differs from the general government expenditures by COFOG (Classification of the Functions of Government) function^r^. The SHA/COFOG differences highlight the uncertainty of estimates due to differences in information sources.

With these limitations in mind, we recommend expanding the Eurostat data collection of hospital discharges with secondary diagnoses. Furthermore, the data coverage of the Eurostat database should be improved to obtain more reliable results for the EU member states since the consistency of reporting is indispensable for international benchmarking of budget expenditures across countries [77].

## Conclusion

This study highlighted the need for cross-country comparison of the public expenditures for substance abuse treatment. Despite limitations, this study presents the public spending for illegal drug and alcohol treatment in hospitals of 21 EU member states. The study corroborates other studies that found that public expenditures for alcohol treatment exceed public expenditures for illegal drug treatment. Multiple factors may influence the number of hospital days and the expenditures that come with it. In this respect, we found a strong correlation between GDP per capita and the public expenditures per hospital day. Other factors should be included in the future analysis of public expenditures for substance abuse treatment, such as the drug policy in a given country (in this study, we especially discussed the case of Sweden) and the social norms regarding alcohol consumption (in this study, we especially discussed various Eastern European countries).

## Endnotes

^a^DALY is a metric to determine the burden of disease. Therefore, it takes into account the years of potential life lost (YLL) due to premature mortality and the years of productive life lost (YLD) due to disability.

^b^The number of people with an alcohol use disorder in treatment is estimated by taking into account the prevalence of 11.9 million people with alcohol dependence and the treatment coverage (in- and outpatient) with a minimum of 8.7% and maximum of 10.2% [3].

^c^The analysis of international databases was part of a larger study on public expenditure on drug treatment that the authors conducted for the EMCDDA in 2013 [78].

^d^Eurostat also reports “general government expenditure by function (COFOG)”. However the COFOG database can only provide data for 19 EU member states (instead of 21 EU member states with the SHA database), since there are no data for Belgium, Spain, Romania and Slovakia. Furthermore, this database does not make a distinction between expenditures for general hospitals, mental health and substance abuse hospitals and specialty hospitals (other than mental health and substance abuse hospitals). Consequently, it would not be possible to estimate hospital expenditures for general and specialty hospitals in Denmark, France, Luxembourg and Netherlands.

^e^The specialty hospitals consists of acute care hospitals; emergency centers; orthopedic hospitals or specialty sanatoriums primarily engaged in providing medical post-acute care, rehabilitative and preventive services; traditional medicine hospitals; and special hospitals for infectious disease (tuberculosis hospitals, hospitals for tropical diseases).

^f^The International Classification of Diseases (ICD) is the international coding system of diseases and other health problems. This standard diagnostic tool is used for epidemiology, health management and clinical purposes.

^g^The European Union reached its current size of 28 member countries with the accession of Croatia on 1 July 2013. Since the analysis is based on 2010 data, Croatia is not included in this study.

^h^The authors also tested an extrapolation by regression. However, the regression with hospitals days for substance abuse treatment regressed on GDP per capita and prevalence of problem substance use was not significant (P > 0.05).

^i^The health expenditures are indispensable to estimate the cost per hospitalization day. An extrapolation of the health expenditures by population would neglect the strong correlation between GDP and hospital expenditures.

^j^Sweden (1507 euros), Austria (1259 euros), Finland (904 euros) and Germany (893 euros) have the highest hospital expenditures per capita.

^k^All means in Tables 1, 2, 3 and 4 are calculated with the simple average method.

^l^Figures 1 and 2 give an impression of the relationship between prevalence and hospital days for substance abuse. This comparison should be interpreted with caution, since hospital days are limited to primary diagnosis of mental and behavioral disorders due to psychoactive substance use or alcohol use.

^m^The data of 15 EU member states are used for extrapolation, because these countries provided data to estimate the public expenditures for all hospital types.

^n^Wittchen et al. [79] state that only 25% of persons with mental disorders receive professional mental health treatment.

^o^The TDI is a monitoring tool developed by the EMCDDA to gain insight into the characteristics, risk behaviors and drug use patterns of people with illegal drug problems. To this end, data are collected on the number and profile of clients entering drug treatment during each calendar year. This tool is being used by 30 countries (28 EU member states, plus Norway and Turkey) who send their national data to the EMCDDA.

^p^The inpatient centers include therapeutic communities, private clinics, units in a hospital and centers that offer residential facilities.

^q^This proportion should be interpreted with caution since the data coverage of TDI ranges from 14% to 100% of existing inpatient units in the registering countries.

^r^The COFOG is restricted to government administrative sources and focuses on the classification of transactions in government-funded health care [80]. The COFOG hospital services expenditures (code GF0703) of 13 EU member states are compared to the SHA hospital expenditures (code HP1). The COFOG expenditures deviate from the expenditures reported on by the SHA: a difference of less than 10% in health expenditures is retrieved for 6 EU member states (Bulgaria, Czech Republic, Germany, Cyprus, Lithuania and Slovenia) and more than 10% for 7 EU member states (Latvia, Austria, Poland, Finland, Hungary, Portugal and Sweden). For Portugal, the hospital expenditure measured by COFOG (749 million euros) is only 12.8% of the expenditure collected by SHA (5.843 million euros).

## Competing interests

The authors declare that they have no competing interests.

## Authors’ contributions

DL conducted the data collection, analysis and drafting of the manuscript. FVD and JC participated in analysis, interpretation, and manuscript revisions. All authors read and approved the final manuscript.

## Supplementary Material

Additional file 1 **Percentage of health expenditures financed by the general government** General government share of total current health expenditure for 21 EU countries, 2010*. *Data for 2010, except for Bulgaria (2008), Cyprus (2008), Latvia (2009) and Luxembourg (2008). Click here for file
