# Supplementary material for: Public spending for illegal drug and alcohol treatment in hospitals: an EU cross-country comparison
Source: Subst Abuse Treat Prev Policy. 2014 Jun 30;9:26. doi: 10.1186/1747-597X-9-26 (PMC4083336; doi:10.1186/1747-597X-9-26)
Supplement: Additional file 1 — Percentage of health expenditures financed by the general government General government share of total current health expenditure for 21 EU countries, 2010*. *Data for 2010, except for Bulgaria (2008), Cyprus (2008), Latvia (2009) and Luxembourg (2008). [file 1747-597X-9-26-S1.pdf]

Additional files

Additional file 1 – Percentage of health expenditures financed by the general government
General government share of total current health expenditure for 21 EU countries, 2010*

Country	Public spending (%)
Austria	77.09
Belgium	75.09
Bulgaria	56.21
Cyprus	42.12
Czech Republic	83.33
Denmark	84.56
Finland	74.39
France	77.46
Germany	77.22
Hungary	64.28
Latvia	59.65
Lithuania	71.48
Luxembourg	82.42
Netherlands	86.09
Poland	71.68
Portugal	67.38
Romania	79.92
Slovenia	73.09
Slovakia	67.82
Sweden	81.51
Spain	73.87
**Mean (SD)**	**72.70 (10.57)**

*Data for 2010, except for Bulgaria (2008), Cyprus (2008), Latvia (2009) and Luxembourg (2008).
